# Composite Electrolyte & Electrode Membranes for Electrochemical Energy Storage & Conversion Devices

**DOI:** 10.3390/membranes10110359

**Published:** 2020-11-21

**Authors:** Giovanni Battista Appetecchi

**Affiliations:** ENEA (Italian National Agency for New Technologies, Energy and Sustainable Economic Development) Materials and Physicochemical Processes Technical Unit (SSPT-PROMAS-MATPRO), Via Anguillarese 301, S. Maria di Galeria, 00123 Rome, Italy; gianni.appetecchi@enea.it; Tel.: +39-06-3048-3924

## 1. Introduction

Currently, electrolyte membranes are largely employed, due to their intriguing peculiarities, as separators in electrochemical energy storage systems such as flow batteries, fuel cells, electrolyzers, etc., and they are also finding applications in commercial batteries and supercapacitors. For instance, one of the most promising approaches for improving safety and reliability of commercial lithium-ion batteries is the adoption of ionically conducting polymer membranes, because of their capability to reduce/minimize the (organic) liquid leakage within the device. Large effort is currently devoted towards optimization and improvement of the chemistry (polymer, salt, solvent, additive, etc.) and formulation of the electrolyte separator as it plays a key role in determining performance and safety of the electrochemical system. Similarly, the formulation of electrodic membranes, which contain passive components such as electronic and/or ionic conductors, polymer binders, etc. not affecting the energy density of the device, strongly influence, however, a device’s power density, cycling behavior and reliability. Therefore, although well-known over time, these issues are currently under deep worldwide investigation and represent a key point for the development of an improved electrochemical system.

The goal of the present Special Issue is presenting a variegated and a detailed overview of the latest findings and frontier approaches regarding these challenging topics. Also, it represents an optimal site for welcoming the latest innovations obtained by key laboratories presently involved. The contained articles cover the following highlights: (i) gel electrolyte membranes for lithium battery systems; (ii) solvent-free electrolyte membranes for lithium battery systems; (iii) electrolyte membranes for fuel cells; and (iv) composite electrode membranes for lithium battery systems. A brief descriptive summary of the scientific contributions is reported here.

## 2. Special Issue Highlights

### 2.1. Gel Electrolyte Membranes for Lithium Battery Systems

Lithium-ion battery systems are one of the best, if not the best, power sources because of their much higher energy density and cycling performance with respect to other cell technologies. However, they use flammable and volatile organic electrolytes, which represent major safety and reliability concerns, leading to dangerous events (thermal runaway) such as venting, fire and cell bluster. A strategy for increasing their safety level is represented by the confinement of the organic electrolyte within proper polymer hosts for obtaining gel Li^+^-conducting membranes able to combine mechanical characteristics of solids (retention of organics) with ion transport properties typical of liquid solutions. Several contributions to this Special Issue focus on this topic.

The manuscript by Barbosa et al. [1] offers a review about recent advancements on different families (homopolymers, co-polymers, composites, and polymer blends) of lithium battery electrolyte separators based on poly(vinylidene fluoride), PVdF. For instance, the separator membrane plays a key role on the battery performance and cycle life, i.e., allowing fast Li^+^ transport and, at the same time, good compatibility towards both electrodes. In particular, the authors highlight the importance of parameters such as polarity and porosity on the properties of the electrolyte. Finally, a comparison with different types of separator membranes is given.

Several compounds were proposed as additives and/or co-components aiming to improve the characteristics (physicochemical/electrochemical/mechanical properties) of gel electrolyte systems. In the present Special Issue, different approaches (even in terms of technological advances) are presented and discussed by the authors.

Navarra et al. [2] report a physicochemical and electrochemical investigation on a composite gel polymer electrolyte (GPE) based on high molecular weight poly(ethylene oxide), PEO, and polymer host reinforced with nanosized silica. The PEO-SiO_2_ blend was obtained by the electrospinning technique, allowing production of the composite polymer host as composed by entangled micro-fibers able of housing/retaining the liquid electrolyte. It was then gelled in a mixed solution formed by an organic solvent-ionic liquid-lithium salt solution. Ionic liquids (ILs), i.e., salts molten at room temperature or below, are non-volatile and non-flammable compounds which are proposed as alternative solvents (in place of the organic ones) for enhancing the safety and reliability of lithium batteries.

Simari et al. [3] propose the incorporation of organo-modified montmorillonite clays as nano-additives into a GPE system constituted by poly(acrylonitrile)/poly(ethylene oxide), PAN/PEO, blend (polymer host), lithium salt and organic solvents (ethylene carbonate/propylene carbonate mixture). The organo-clays were prepared by intercalation of CTAB molecules in the interlamellar space of sodium smectite clay through a cation-exchange reaction. A multinuclear NMR spectroscopy investigation allowed measuring the ^7^Li and ^19^F self-diffusion coefficients, and the spin-lattice relaxation times. Additionally, a full description of the ions dynamics is reported (including ion transport number and ionicity index).

The effect of ionic liquid addition into GPEs is reported and discussed also in the contribution by Villarreal et al. [4], who have been investigating a mixed organic/ionic liquid electrolytic system. In addition, the authors studied the beneficial effect of incorporation of succinonitrile additive (5 wt.%) on the GPE ion transport properties and on the cycling performance of Li/SnO_2_-C and Li/LiCoO_2_ polymer half-cells.

### 2.2. Solvent-Free Electrolyte Membranes for Lithium Battery Systems

Polymer-salt systems are liquid-free, solid-state solutions (namely solid polymer electrolytes, SPEs) in which the polymer acts as the solvent and the salt (LiX) is the solute. A typical example is represented by the PEO-LiX complexes. The results of development of SPEs are appealing from the safety and technological (i.e., possibility to be manufactured, easily and at low-cost, into thicknesses and shapes not allowed by liquid electrolytes, and better flexibility and mechanical robustness) points of view, thus opening new perspectives to applications in lithium batteries. The absence of any liquid (organic) leakage remarkably enhances the safety and reliability of the electrochemical device and substantially improves the interfacial behavior; these advantages could make possible the employment of lithium metal anodes.

The manuscript by Munoz et al. [5], highlighting how polymer electrolyte development is still a priority, gives a wide review aimed at illustrating various approaches to PEO SPEs, using the NMR spectroscopy technique for investigating their chemical structure and ion transport properties.

PEO electrolyte systems are known to exhibit ion conductivity values suitable for practical applications only at medium-high temperatures (above 70 °C, at which PEO is fully amorphous); this considerably narrows the operative temperature range of all-solid-state lithium polymer batteries. Therefore, a large effort was devoted to prevent PEO host crystallization, thus extending the amorphous phase content at lower temperatures. A very promising approach for improving the ion transport properties of SPEs is represented by the incorporation of ionic liquids into the polymer matrix.

The contributions by González et al. [6] and Kim et al. [7] focus on an IL-containing, thermoplastic PEO electrolyte, prepared through solvent-free processes, to obtain solid, Li^+^-conducting membrane separators. In addition, González et al. proposed a reinforcement of the SPE system with modified sepiolite (TPGS-S). The authors demonstrate how the incorporation of the ionic liquid largely improves the thermal, ion-transport and interfacial properties (particularly with the lithium metal anode) of the polymer electrolyte. Tests carried out in all-solid-state cells have shown excellent cycling performance and capacity retention, even at high rates, which are never tackled by ionic liquid-free SPEs.

### 2.3. Electrolyte Membranes for Fuel Cells

Electrochemical energy conversion devices as fuel cells are rather attractive for automotive and stationary applications. The core of this clean technology is the electrolyte separator, typically a Nafion (sulfonate tetrafluoroethylene-based copolymer) proton exchange membrane. For more efficient power generation, fuel cells should operate above 80 °C at which temperature, however, Nafion suffers from water evaporation, leading to remarkable proton conductivity decay. One approach for overcoming this drawback is represented by the incorporation of inorganic additives into Nafion membranes in order to improve the water retention.

In the frame of the present Special Issue, Mazzapioda et al. [8] report a physicochemical investigation into composite proton-conducting membranes for fuel cell applications, based on a Nafion host and incorporating a calcium titanium oxide (CaTiO_3−δ_) filler. The authors discuss how the addition of the additive, besides enhancing the mechanical properties, improves the water affinity and ionic conductivity. However, high filler contents are seen to play a detrimental effect.

### 2.4. Composite Electrode Membranes for Lithium Battery Systems

The fourth highlight of this Special Issue is dedicated to composite electrode membranes to be addressed by electrochemical energy devices, in particular lithium batteries. The scientific and technological community involved in this field knows very well the importance of the electrode formulation, i.e., ability of allowing high electronic conductivity and sufficient porosity in combination with good mechanical properties, on the overall battery behavior [1]. At the same time, even the processing route plays a decisive role in determining the electrode performance, resulting in primary importance for certain application. Among these, printed batteries are receiving increasing interest due to the daily growing use of small-size electronic devices, which require very thin-layer batteries for the energy supply. Having this target in mind, industrial gravure printing can represent a proper production technology due to its high speed and quality, and its capability to realize whatever shape electrodes.

The contribution by Montanino et al. [9] proposes the realization of LiFePO_4_ cathode tapes through gravure printing. This technique, even if it is one of the most appealing for realizing functional layers, has not been investigated in detail until now. In addition, the authors have explored the possibility of employing the printing technique for battery manufacturing.

## 3. Final Remarks

To summarize, the increasing need of more and more performant (in terms of energy and power density, cycle life and safety) electrochemical devices for storage and conversion of energy is strongly pushing towards the formulation of the new electrolytes. The research on electrolyte separators capable of granting lower cost, safer, more benign and environmentally friendly cells will also expand in view of a large-scale diffusion of hybrid/electric vehicles and delocalized small stationary storage systems. For instance, the electrolyte membrane formulation is expected to play a key role in the cell performance rating capability, compatibility with electrodes, cycling behavior and safety. Also, a deeper understanding of the polymer electrolyte restricted inside the electrode pores is needed for a proper formulation design. In the frame of this scenario, the present Special Issue aims to offer an overview about the different approaches followed to achieve the above-mentioned targets, in particular in the field of electrolyte membranes.
